# Dietary supplementation with fermented Mao-tai lees beneficially affects gut microbiota structure and function in pigs

**DOI:** 10.1186/s13568-019-0747-z

**Published:** 2019-02-18

**Authors:** Huan Li, Huawei Li, Peifeng Xie, Zhihua Li, Yulong Yin, Francois Blachier, Xiangfeng Kong

**Affiliations:** 1 Hunan Provincial Key Laboratory of Animal Nutritional Physiology and Metabolic Process, Key Laboratory of Agro-ecological Processes in Subtropical Region, National Engineering Laboratory for Pollution Control and Waste Utilization in Livestock and Poultry Production, Institute of Subtropical Agriculture, Chinese Academy of Sciences, Changsha, Hunan 410125 China; 2 Institute of Occupational Health and Environmental Health, School of Public Health, Lanzhou University, Lanzhou, Gansu 730000 China; 3 UMR PNCA, AgroParisTech, INRA, Université Paris-Saclay, Paris, France

**Keywords:** Gut microbiota, Fermented Mao-tai lees, Short-chain fatty acids, Beta diversity, Beneficial bacteria, Livestock farming

## Abstract

Gut microbiota positively contribute to livestock nutrition and metabolism. The manipulation of these microbes may improve animal health. Some feed additives improve livestock health and metabolism by regulating gut microbiota composition and activity. We fed hybrid pigs diets supplemented with 0% (control), 5% (treat 1), 10% (treat 2), or 15% (treat 3) fermented Mao-tai lees (FML) for 90 days. Short-chain fatty acids (SCFAs), bioamines, and microbial communities found in colonic contents were analyzed to investigate microbiota composition and metabolic profiles. Concentrations of straight-chain fatty acids (e.g., acetate, propionate, and butyrate) and tyramine increased with FML supplementation content. Contrary to the minor effects of 5% and 10% FML on gut microbiota, 15% FML influenced beta diversity (Jaccard or Bray–Curtis dissimilarity) but not alpha diversity (number of operational taxonomic units and Shannon diversity) of pig gut microbial communities compared to the control group. Notably, 15% FML animals were characterized by a higher abundance of potentially beneficial bacteria (*Lactobacillus* and *Akkermansia*) but lower abundances of potential pathogens (*Escherichia*). Numerous genes associated with metabolism (e.g., starch, sucrose, and sulfur-compounds metabolism) showed a higher relative abundance in the 15% FML than in the control group. Additionally, most *Phascolarctobacterium*, *Treponema*, *Prevotella*, and *Faecalibacterium* bacterial markers in the 15% FML group were positively correlated with straight-chain fatty acid concentrations, suggesting that these bacteria are likely associated with SCFA production. Taken together, our findings demonstrate the beneficial effects of 15% FML on fermentation of undigested compounds and gut microbiota composition in the colon. Thus, 15% FML supplementation in pig feed may possibly represent a way to optimize pig colon health for livestock farming.

**Electronic supplementary material:**

The online version of this article (10.1186/s13568-019-0747-z) contains supplementary material, which is available to authorized users.

## Introduction

Healthy livestock farming technologies and management practices are a central concern in animal farming industry, which must provide high-quality meat products to safely meet human consumption requirements. In particular, pigs are one of the most important economic livestock species in numerous countries. Pigs represent the largest livestock product in China, with great commercial and economic values worldwide (Bai et al. 2014; Sun and Jia 2015; Yu and Abler 2014). However, intensive or large-scale livestock farming causes various problems such as serious feed resource shortages and grain competition between humans and livestock (Ilea 2009). In addition, the utilization of antibiotics in livestock husbandry potentially poses a threat to livestock health, and it could lead to environmental pollution as well as a decline in animal immunity, gut microbiota disorders, and an increased risk of the spread of antibiotic-resistant bacteria genes in the environment (Baquero et al. 2008; Wu et al. 2010; Zhao et al. 2018). Residues of antibiotics in livestock products are likely harmful to human health (Marshall and Levy 2011). However, the restricted use of antibiotics in feed used in livestock farming may increase disease rates in animals and decrease amounts of global products. Therefore, it is imperative to develop safe and effective feed additives that could replace antibiotics, thus improving livestock health and products. Recently, studies focused on replacing antibiotics in livestock husbandry have examined the effects of probiotics, prebiotics, synbiotics, and other dietary additives on livestock (Abudabos et al. 2017; Markowiak and Slizewska 2018; Marshall and Levy 2011). Moreover, these studies have aroused the interests of scientists globally, since these newly developed feed supplements were found to improve livestock health and growth (Markowiak and Slizewska 2018).

Gut microbiota has several beneficial functions in hosts, including food digestion, energy harvesting, immune regulation, and resistance against pathogens (Rooks and Garrett 2016; Stanley et al. 2016; Tremaroli and Bäckhed 2012). Evidence suggests that probiotics and prebiotics could beneficially modify gut microbiota composition and activity in humans and animals (Barba-Vidal et al. 2018; Moura et al. 2007), thereby improving host metabolism and health, notably at the intestinal level. For example, the probiotics *Lactobacillus* (*Lactobacillus reuteri avibro*) and *Bacillus* (*Bacillus subtilis* and *Bacillus licheniformis*) exhibited the ability to increase nutrient digestibility and animal performance, and they reduced the abundance of pathogens (*Salmonella* and *Escherichia coli*) (Ahmed et al. 2014). In pigs, some prebiotic products, such as xylo-oligosaccharides (XOS), β-mannanase, mannan-oligosaccharides, and yeast cultures have been used to modify gut microbiota composition, improve host immune response, stimulate the growth of more beneficial bacteria, and inhibit the colonization or abundance of pathogens by producing antimicrobial substances in humans and livestock (Barros et al. 2015; De Maesschalck et al. 2015; Liu et al. 2018; Rastall and Gibson 2015). For instance, in broiler chicken, XOS feed additives modulate the gut microbiota and increase the abundance of the potential beneficial bacteria *Lactobacillus* and *Bifidobacterium* (Pourabedin et al. 2015). Moreover, feed containing yeast cultures increased the abundance of *Clostridium* and *Methylobacterium*, which are important cellulose-degrading bacteria that may help herbivorous grass carp (*Ctenopharyngodon idellus*) to degrade ingested plant-based food (Liu et al. 2018).

Recently, liquor lees were been used as promising feed additive candidates, and they were found to be able to shape animal gut microbiota. For example, the content of dried distiller grains with solubles (DDGS) in feed is positively correlated with gut microbial diversity in birds (Abudabos et al. 2017), and increased microbial diversity is associated with metabolic activity and health in human and animal hosts (Li et al. 2017b, 2018; Tap et al. 2015). However, studies of the effects of liquor lees on livestock gut microbiota are still in their infancy and need further development.

Guizhou Maotai liquor is a fragrant and tasty wine (Wu et al. 2013), and it is renowned as the best Chinese sauce fragrance liquor. The production of Maotai wine was about 38,700 t, and the solid by-products (including lees) were about 110,000 t (Li et al. 2015). DDGS is a suitable substrate for solid-state fermentation (SSF), and only moderate changes were found in its nutritional profile after SSF (Yang et al. 2012). Fermented Maotai lees (FML) also have a high nutritional value (e.g., high protein, cellulose, amino acids, and organic acids), so they may be of interest for livestock farming. Here, we used MiSeq sequencing of 16S rRNA genes to evaluate the effects of FML on the gut microbiota diversity and also measured the gut metabolic products (short-chain fatty acids (SCFAs) and bioamines) in pigs. We solved three questions: (1) Do FML influence the SCFA and bioamine pofiles in the pig gut? (2) Whether FML modify gut microbiota composition and function? (3) Do FML increase the abundance of potential beneficial bacteria and decrease the abundance of specific pathogens?

## Materials and methods

### Experimental design and ethical standards

A total of 24 Duroc × Large White × Landrace hybrid barrows (male), with an initial body weight (BW) of 42.28 ± 1.23 kg (mean ± SE), were fed a corn and soybean meal-based diet (Additional file 1: Table S1), which met the National Research Council (NRC 2012) requirements for growing-finishing pigs. After these pigs were fed the same diet for 1 week, the animals were randomly arranged to one of the four treatments. Each treatment group had 6 replicates. Pigs in the control group were fed a basic diet, whereas those in the experimental groups were fed the basic diet containing 5%, 10%, or 15% fermented Maotai lees (FML) [0% as Control, 5% (treat 1), 10% (treat 2) and 15% (treat 3)] during the experimental period. Notably, the FML used in the present study were provided by the Road Biological Environmental Co., Ltd., Sichuan, China. The FML products were fermented by saccharomyces yeasts with acid-resisting, overproducing enzyme, and high vigor. After fermentation, the mixture was dried at 60 °C using laboratory incubator, and then smashed. The FML were then stored in − 20 °C for experimental use. The determined nutrient levels (%) of the FML based on dry matter content (92.97%) were as follows: ash, 9.28; gross energy (GE), 18.29; crude protein (CP), 23.96; ether extract (EE), 5.39; crude fiber (CF), 17.67; acid detergent fiber (ADF), 38.06; neutral detergent fiber, 47.28; Ca, 0.53; P, 0.55.

Experimental pigs were housed in cages (3.5 m × 5.0 m) equipped with feed intake recording equipment (Beijing Hamoer Automation Equipment Co., Ltd., Beijing, China). The space provided by the equipment allows one pig at a time to have ad libitum access to the diet. Animals had 24 h access to feed and water during the whole course of the experiment. Pigs were labeled with an individual electronic ear marker. The feeding trial lasted for 90 days. The final body weight of each pig was also measured to evaluate the growth status.

The experimental design and procedures in this study were reviewed and approved by the Animal Care and Use Committee of the Institute of Subtropical Agriculture, Chinese Academy of Science. Processing of animal experiments and sample collection strictly followed the relevant guidelines.

### Sample collection

At the end of the feeding trial, the diet was removed 12 h before slaughter. All pigs were transported from the farm to the processing facility (approximately 40 km) at 7:00 h, and sacrificed at 19:00 h under commercial conditions using electrical stunning (120 V, 200 Hz). Colonic contents were collected into 50 ml sterile tubes. After complete mixing, all the gut contents were stored at − 20 °C for the following microbiota and metabolic analysis.

### Measurements of short-chain fatty acids (SCFAs) and bioamines

Gut SCFAs (mg/g), including straight-chain fatty acids (acetate, propionate, butyrate, and valerate) and BCFAs (branched-chain fatty acid, including isobutyrate and isovalerate) were analyzed using gas chromatography as previously described (Ji et al. 2018). Bioamines (μg/g), including putrescine, cadaverine, tyramine, spermidine, and spermine were measured using high-performance liquid chromatography as previously described (Ji et al. 2018).

### DNA extraction and MiSeq sequencing of microbial 16S rRNA gene

Genomic DNA of colonic contents was extracted using a QIAamp DNA Stool Mini Kit (Qiagen, Hilden, Germany) according to the manufacturer’s instructions. The DNA concentration of each sample was measured with a NanoDrop^®^ ND-1000 instrument (NanoDrop Technologies Inc., Dover, USA). The extracted DNA was diluted to 10 ng/µl for the following polymerase chain reaction (PCR) amplification. The protocols of PCR amplification, gel extraction, and sequencing library construction were described previously (Li et al. 2016a, b, c). The universal primers 338F (5′-ACTCCTACGGGAGGCAGCAG-3′) and 806R (5′-GGACTACHVGGGTWTCTAAT-3′) with 12 nt unique barcode at 5′-end of 338F were used to amplify the V3-V4 region of bacterial 16S rRNA gene (Jiang et al. 2018). After PCR amplification, amplicons were extracted from 1.2% agarose gels and purified using SanPrep DNA Gel Extraction Kit (Sangon Biotech, Shanghai, China) and quantified with a NanoDrop^®^ ND-1000 instrument (NanoDrop Technologies Inc., Dover, USA). Purified amplicons were mixed in equal molar together and underwent paired-end sequencing using an Illumina MiSeq sequencer (MiSeq Reagent Kit V.2, California, USA, 500 cycles).

### Bioinformatics analysis

Bioinformatics analysis followed the analytical methods of Li et al. (2016b). Briefly, the raw sequences were analyzed using QIIME Pipeline-Version 1.9.0 (http://qiime.org/tutorials/tutorial.html) (Caporaso et al. 2010). All sequences were trimmed and assigned to each sample based on their unique barcodes only if they completely matched their unique barcodes. The FLASH-1.2.8 software (Caporaso et al. 2012) was used to merge the overlapping paired-end reads. The merged sequences with high quality (reads length more than 300 bp, without ambiguous base “N”, and average base quality score more than 30) were kept for further analysis. Then the aligned 16S rRNA gene sequences underwent a chimera check using the Uchime algorithm (Edgar et al. 2011). Due to possible contamination of chloroplast sequences in PCR amplification, the Metaxa2 software tool was used to remove chloroplast sequences from our large sequencing datasets (Bengtsson-Palme et al. 2015). After filtering chimeras and chloroplasts, the remaining sequences were clustered into operational taxonomic units (OTUs) at a 97% identity threshold with an open-reference OTU picking method using the Uclust algorithm (Edgar 2010). Those sequences not classifying to bacteria (*Eukaryota* and *Archaea* lineages) were filtered out. Singleton OTUs were also removed. The most abundant sequences within each OTU were defined as “representative sequences”. Taxonomic classification of representative sequences was implemented using the Ribosomal Database Project classifier in the QIIME platform (Wang et al. 2007).

To minimize the impact of uneven sequencing depth, each sample was rarefied to 32,805 sequences. To evaluate alpha diversity indices, Goods coverage, Chao1, observed OTUs, Shannon diversity and evenness were calculated. The rarefaction curves were generated from the observed OTUs at the OTU level. To assess beta diversity, Jaccard and Bray–Curtis distance metrics were produced through the QIIME pipeline. Jaccard distance is based on the presence/absence of OTUs/species (Jaccard 1912), whereas Bray–Curtis distance is based on the both OTU abundance and presence/absence (Bray and Curtis 1957). Differences in overall bacterial community structure among groups were visualized using the non-metric multidimensional scaling (NMDS) plots of the two dissimilarity matrices.

### Statistical analysis

Permutational multivariate analysis of variance (PERMANOVA) was used to reveal whether the structures of gut microbiota were significantly different among groups based on the Jaccard and Bray–Curtis distance matrices using “adonis” in the R ‘vegan’ package (Li et al. 2017a). The model also comprised other variables, including animal body weight. Taxonomic profiles were evaluated at the phylum and genus levels. Differences in relative abundances of the genera were performed through the *group significance.py* script in the QIIME platform with one-way analysis of variance (one-way ANOVA) with Tukey’s post hoc test. *P*-values were corrected using false discovery rate (FDR). Comparison of the alpha diversity indices among groups was tested using Mann–Whitney *U* tests. Statistical significance was accepted at a *P* < 0.05. Linear discriminant analysis effect size (LEfSe) (Nicola et al. 2011) was used to identify bacterial OTU biomarkers at OTU level among groups based on a *P* < 0.05 and LDA score > 2.0. This analysis was performed online in the Galaxy workflow framework (http://huttenhower.sph.harvard.edu/galaxy/).

The Spearman correlation analysis among SCFA levels, bioamines, bacterial biomarkers (based on LefSe results) was achieved using function “cor.test” of the package “Stats” in R. One heatmap plot of the correlation values were produced using package “gplots”. One-way ANOVA with Tukey’s post hoc test was used to evaluate the difference of SCFA levels and bioamines among groups. Absolute SCFA or bioamine concentrations (μmol/g) were converted to molar percentages within each individuals (Li et al. 2018), and then differences in overall SCFA or bioamine profile were visualized using the NMDS plots of Bray–Curtis dissimilarity indices. PERMANOVA was also used to reveal whether the structures of SCFA and bioamine profiles were significantly different among groups based on the Bray–Curtis distance matrices using “adonis” in the R ‘vegan’ package.

### Predicted metagenomes

PICRUSTv1.0.0 (Langille et al. 2013) was used to predict abundances of functional gene from OTU abundances based on the 16S rRNA gene sequences. We only focused on the gene functions associated with metabolism between the control and treat 3 groups. Two-tailed *t* tests (Bonferroni-corrected) were performed to test the differences of gene functions between these two groups.

### Nucleotide sequence accession numbers

The original 16S rRNA data were available at the European Nucleotide Archive by accession number PRJEB27667 (http://www.ebi.ac.uk/ena/data/view/ PRJEB27667).

## Results

### The effects of FML on the weight gain, gut SCFAs, and bioamines of pigs

**Fig. 1:**
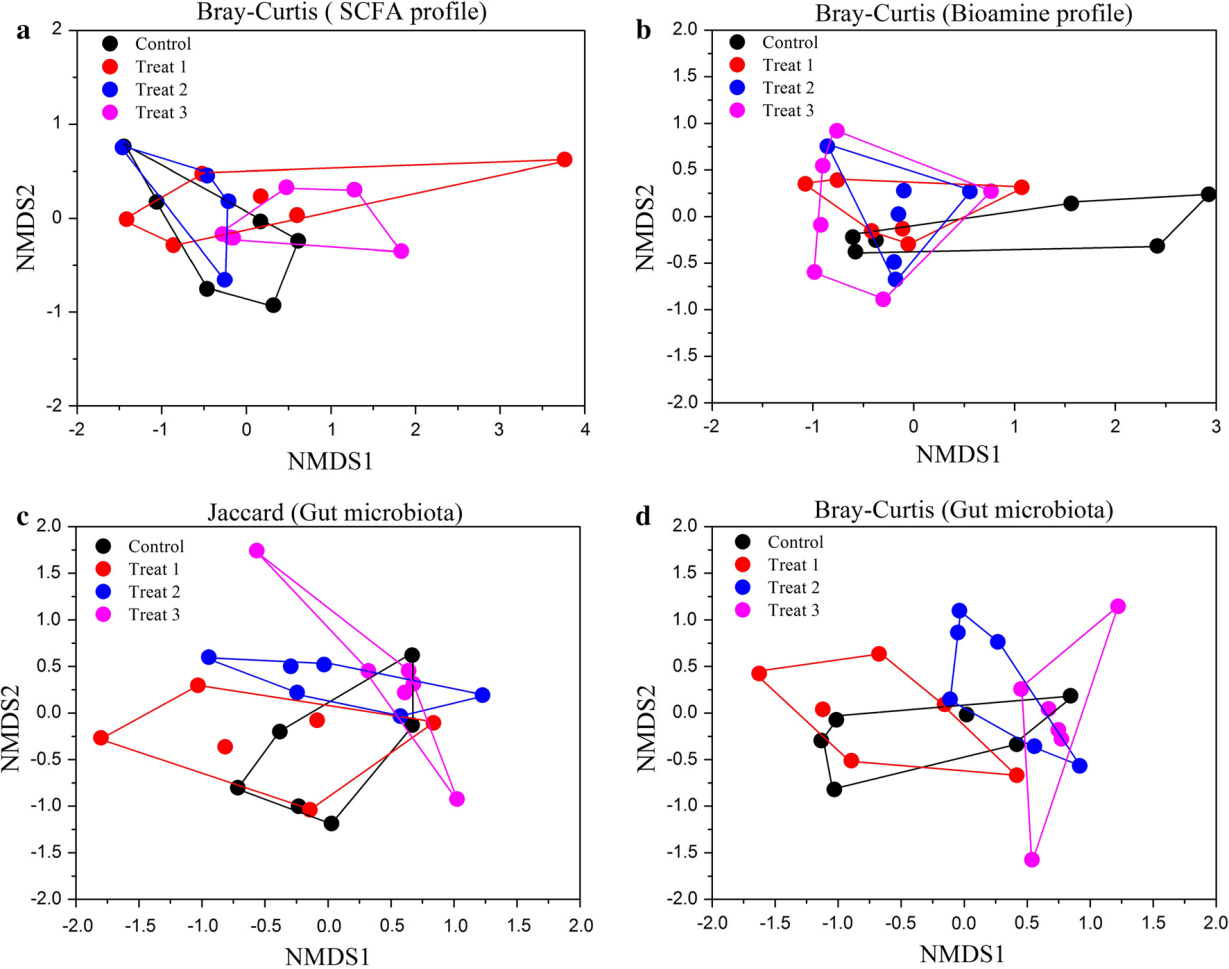
NMDS plots of dissimilarity metrics comparing the profiles of gut microbiota, SCFAs and bioamines among groups. **a** SCFA profile based on Bray–Curtis dissimilarities. **b** Bioamine profile based on Bray–Curtis dissimilarities. **c** Gut microbiota structure based on Jaccard dissimilarities. **d** Gut microbiota structure based on Bray–Curtis dissimilarities

Dietary FML supplementation had no significant effects on the weight gain of pigs (Table 1). However, the concentrations of acetate, propionate, butyrate, valerate, total straight-chain fatty acids, and total SCFAs in the gut contents significantly increased with FML content in the feed (Spearman correlation analysis, all *P* < 0.05; Table 1), whereas there were no significant differences in BCFA (including isobutyrate, isovalerate, and total BCFA) concentration among groups. Over SCFA profile structure showed no significant separation between control and treat groups (Fig. 1a; Additional file 1: Table S2; PERMANOVA, all *P* > 0.05). However, final body weight had significant influences in shaping the SCFA profile (PERMANOVA, *R*^2^ = 0.149, *P* = 0.027).

**Table 1:** The comparison of body weight, fecal short chain fatty acids (SCFAs), and bioamine concentrations in pigs with fermented Mao-tai lees (FML) contents

Items	Control	Treat 1	Treat 2	Treat 3
*Body weight (kg)*				
Initial Body weight	46.250 ± 3.080a	38.620 ± 1.990a	42.750 ± 2.620a	41.480 ± 1.310a
Final body weight	111.280 ± 5.660a	108.120 ± 4.330a	112.950 ± 4.100a	106.750 ± 3.980a
Weight gain	65.030 ± 3.450a	69.500 ± 3.360a	70.200 ± 3.240a	65.270 ± 3.620a
*SCFAs (mg/g)*				
Acetate	4.097 ± 0.565ab	4.256 ± 0.271b	5.813 ± 0.071a	6.199 ± 0.871ab
Propionate	2.012 ± 0.289a	2.066 ± 0.260a	2.833 ± 0.107a	2.670 ± 0.319a
Isobutyrate	0.115 ± 0.022a	0.163 ± 0.011a	0.151 ± 0.023a	0.171 ± 0.029a
Butyrate	1.514 ± 0.289a	1.436 ± 0.221a	2.256 ± 0.154a	1.890 ± 0.184a
Isovalerate	0.202 ± 0.038a	0.302 ± 0.023a	0.274 ± 0.041a	0.313 ± 0.052a
Valerate	0.172 ± 0.022a	0.237 ± 0.026ab	0.307 ± 0.018b	0.262 ± 0.021b
Total straight-chain fatty acids	7.622 ± 1.120a	7.758 ± 0.727a	10.903 ± 0.315a	10.760 ± 1.307a
Total BCFA	0.317 ± 0.060a	0.465 ± 0.034a	0.425 ± 0.064a	0.484 ± 0.081a
Total SCFA	7.940 ± 1.176a	8.223 ± 0.709a	11.327 ± 0.366a	11.244 ± 1.340a
*Bioamines (μg/g)*				
Putrescine	5.259 ± 1.065a	4.322 ± 0.499a	4.855 ± 0.470a	3.362 ± 0.691a
Cadaverine	11.140 ± 5.438a	4.188 ± 1.811a	4.079 ± 0.991a	2.667 ± 1.342a
Tyramine	1.013 ± 0.259a	1.400 ± 0.257a	1.294 ± 0.341a	2.082 ± 0.347a
Spermidine	5.584 ± 0.826a	8.746 ± 0.817ab	9.240 ± 0.709b	7.014 ± 1.244ab
Spermine	0.547 ± 0.090a	0.731 ± 0.095a	0.869 ± 0.161a	0.454 ± 0.091a

Data was expressed as mean ± SE obtained from 6 individual pigs each. One-way ANOVA with Tukey’s post hoc test or Mann–Whitney U-tests was used to evaluate the difference of fecal metabolites in different groups. Significant difference is indicated by different letters. The group control, treat 1, treat 2, and treat 3 signify that feed is supplemented with 0, 5%, 10%, or 15% FML, respectively

In addition, the concentrations of most bioamines showed no differences between groups, whereas the spermidine concentration of 10% FML group was significantly higher than that of control group (Table 1). Over bioamine profile structure also showed no significant separation between control and treat groups (Fig. 1b; Additional file 1: Table S2; PERMANOVA, all *P* > 0.05). Final body weight also had no significant impacts in shaping the bioamine profile (PERMANOVA, *P* > 0.05).

### The influences of FML on alpha and beta diversity of pig gut microbiota

A total of 2,394,088 sequences were generated from the 24 pig samples. After filtering out low-quality sequences, chimeras, chloroplasts, singletons, and those sequences not classifying to bacteria, we obtained 922,668 valid sequences. To compare samples with different sequencing depth, each sample was rarefied to 32,805 sequences. At a threshold of 97% sequence similarity, 21,920 unique OTUs were identified using Uclust clustering. The rarefaction curve of observed OTUs across all samples reach stable values (Additional file 1: Fig. S1), indicating that our sequencing had captured most species although additional sequencing may detect some rare OTUs. In addition, the Goods coverage (mean ± SE) of gut microbiota at OTU level across all samples was 95.01% ± 0.26%, confirming that our sequencing depth is enough to detect a majority of gut bacterial species. The alpha diversity values (including Chao1, observed OTUs, Shannon diversity, and evenness) had no significant differences between the control and treat groups (Mann–Whitney *U* tests, all *P* > 0.05; Table 2).

**Table 2:** Alpha diversity values of the gut microbiota were evaluated using OTUs defined at 97% similarity threshold

Items	Control	Treat 1	Treat 2	Treat 3
Goods coverage	0.948 ± 0.004a	0.953 ± 0.005a	0.954 ± 0.005a	0.945 ± 0.007a
Chao1	6049.249 ± 493.238a	5584.114 ± 600.391a	5510.697 ± 578.835a	6693.918 ± 904.801a
Observed OTUs	2974.500 ± 223.060a	2667.667 ± 285.628a	2648.667 ± 249.738a	3184.167 ± 289.860a
Shannon diversity	7.574 ± 0.376a	7.132 ± 0.408a	7.755 ± 0.216a	8.216 ± 0.214a
Evenness	0.657 ± 0.027a	0.627 ± 0.028a	0.684 ± 0.012a	0.707 ± 0.011a

Data was expressed as mean ± SE obtained from 6 individual pigs each. Mann–Whitney U-tests were used to evaluate the difference in different groups. The group control, treat 1, treat 2, and treat 3 signify that feed is supplemented with 0, 5%, 10%, or 15% fermented Mao-tai lees, respectively. Significant difference is indicated by different letters

However, the beta diversity values of gut microbiota had significant differences across groups based on Jaccard (Fig. 1c; Table 3; PERMANOVA, *R*^2^ = 0.057, *P* = 0.003) and Bray–Curtis distance matrices (Fig. 1d; Table 3; PERMANOVA, *R*^2^ = 0.212, *P* = 0.008). When we compared the pairwise dissimilarity matrices between any two groups, our results showed that only the community structure of 15% FML group had significant differences with that of the control group (Table 3) based on Jaccard (PERMANOVA, *R*^2^ = 0.11, *P* = 0.015) or Bray–Curtis distance (PERMANOVA, *R*^2^ = 0.171, *P* = 0.017), indicating that only 15% FML influences the community structure of pig gut microbiota. In addition, the group 15% FML showed different community structures with 5% FML based on Jaccard (PERMANOVA, *R*^2^ = 0.114, *P* = 0.023) or Bray–Curtis distance (PERMANOVA, *R*^2^ = 0.228, *P* = 0.008). However, final body weight had no significant impacts in shaping the beta diversity values of pig gut microbiota (Jaccard, PERMANOVA, *P* > 0.05; Bray–Curtis, *P* > 0.05).

**Table 3:** PERMANOVA showing different community compositions and structures among different groups

Items	Jaccard		Bray–Curtis	
	*R* ^2^	*P*	*R* ^2^	*P*
All	0.057	0.003*	0.212	0.008*
Control vs treat 1	0.090	0.475	0.079	0.554
Control vs treat 2	0.103	0.052	0.135	0.113
Control vs treat 3	0.110	0.015*	0.171	0.017*
Treat 1 vs treat 2	0.099	0.15	0.154	0.053
Treat 1 vs treat 3	0.114	0.023*	0.228	0.008*
Treat 2 vs treat 3	0.099	0.104	0.132	0.066

Significant differences (*P* < 0.05) are indicated by asterisk (*). The group control, treat 1, treat 2, and treat 3 signify that feed is supplemented with 0, 5%, 10%, or 15% fermented Mao-tai lees, respectively

### FML treatments affect the taxonomic composition of gut microbiota

**Fig. 2:**
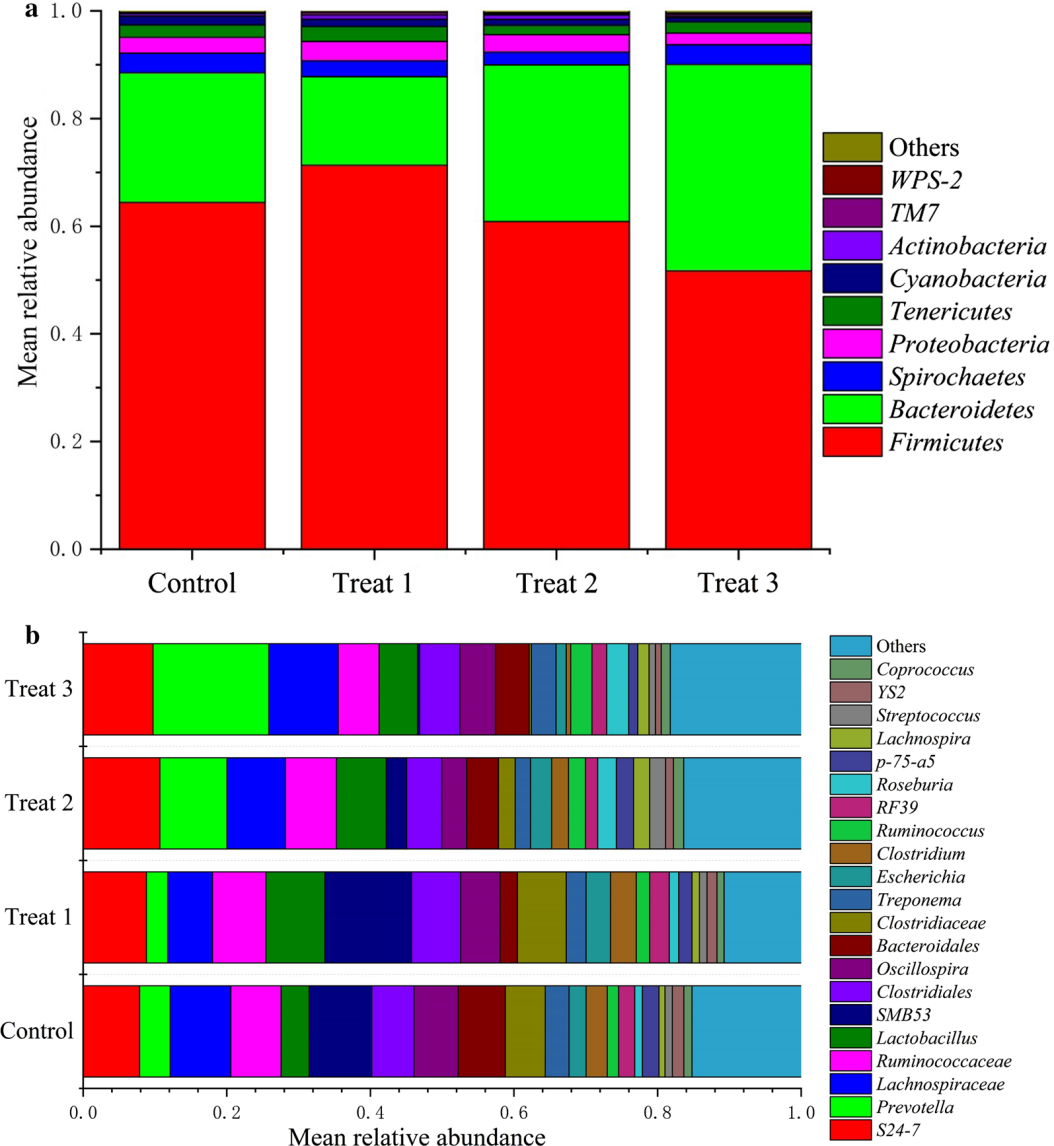
Taxonomic compositions of gut bacterial communities with different dietary treatments with fermented Mao-tai lees in pigs. **a** The relative abundance of bacterial phyla. **b** Their relative abundance of top 21 bacterial taxa within a group at genus level

Across all samples, approximately 99% of the total sequences were assigned into 23 phyla. Among these phyla, Firmicutes (62.1%), Bacteroidetes (27.0%), Spirochaetes (3.2%), Proteobacteria (3.0%), Tenericutes (2.2%), and Cyanobacteria (1.2%) were the six dominant bacterial taxa (mean relative abundance > 1%) in the pig gut. Some rare phyla included Actinobacteria, TM7, and WPS-2. Those phyla with mean relative abundance > 0.1% are shown in Fig. 2a. At genus level, S24-7, *Prevotella*, Lachnospiraceae, Ruminococcaceae, *Lactobacillus*, *SMB53*, Clostridiales, *Oscillospira*, Bacteroidales, Clostridiaceae, *Treponema*, *Escherichia*, *Clostridium*, *Ruminococcus*, RF39, *Roseburia*, *p*-*75*-*a5*, *Lachnospira*, *Streptococcus*, YS2, and *Coprococcus* were the dominant bacterial genera (Fig. 2b).

We further compared the significant difference of bacterial genera among groups. A total of 14 genera showed significant difference among groups (Table 4). Among these genera, the relative abundance of *Roseburia*, *Desulfovibrio*, *Phascolarctobacterium*, *Oxalobacter*, *Defluviitalea,* and *Prevotella* in the group 15% FML was significantly higher than that of the control group (one-way ANOVA, all *P *< 0.05). A total of 8 genera, such as *Butyricicoccus*, *Dorea*, *Clostridium*, *SMB53,* and *Peptococcus*, showed a lower abundance than that of the control group (all *P *< 0.05).

**Table 4:** Comparsion of mean relative abundance of bacterial genera among groups

Genus (%)	Control	Treat 1	Treat 2	Treat 3
*Roseburia*	1.058 ± 0.135a	1.308 ± 0.198a	2.602 ± 0.494ab	3.061 ± 0.621b
*Butyricicoccus*	0.166 ± 0.080a	0.148 ± 0.044a	0.479 ± 0.113b	0.145 ± 0.036a
*Peptostreptococcaceae*_unclassified genus	0.186 ± 0.069ab	0.233 ± 0.065a	0.061 ± 0.013ab	0.016 ± 0.054b
*Dorea*	0.080 ± 0.020ab	0.055 ± 0.017a	0.175 ± 0.049b	0.036 ± 0.016a
*Clostridium*	0.169 ± 0.012a	0.185 ± 0.021a	0.064 ± 0.031b	0.024 ± 0.017b
*Desulfovibrio*	0.023 ± 0.019a	0.007 ± 0.004a	0.02 ± 0.002a	0.245 ± 0.110b
*Phascolarctobacterium*	0.180 ± 0.11a	0.148 ± 0.079a	0.469 ± 0.080ab	0.732 ± 0.218b
*SMB53*	8.737 ± 3.998ab	12.069 ± 3.383a	2.858 ± 1.065ab	0.296 ± 0.051b
*Oxalobacter*	0.002 ± 0.002a	0.006 ± 0.006ab	0.001 ± 0.001a	0.042 ± 0.019b
*Defluviitalea*	0.047 ± 0.018a	0.056 ± 0.030ab	0.143 ± 0.019b	0.067 ± 0.020ab
*Clostridiaceae*_unclassified genus	5.549 ± 2.137a	6.798 ± 2.280a	2.318 ± 0.359b	0.303 ± 0.068b
*Peptostreptococcaceae*_unclassified genus	0.146 ± 0.068a	0.159 ± 0.041a	0.039 ± 0.013a	0.007 ± 0.010a
*Prevotella*	4.264 ± 2.183ab	2.973 ± 0.940a	9.349 ± 1.695ab	16.127 ± 5.704b
*Peptococcus*	0.083 ± 0.069a	0.053 ± 0.019ab	0.023 ± 0.007ab	0.012 ± 0.004b

Only those genera that were significant different genera (*P* < 0.05) among all groups are shown

Data was expressed as mean ± SE obtained from 6 individual pigs each. Significant difference is indicated by different letters between groups. One-way analysis of variance (one-way ANOVA) with Tukey’s post hoc test was used to test the differences. *P*-values were corrected using false discovery rate (FDR). The group control, treat 1, treat 2, and treat 3 signify that feed is supplemented with 0, 5%, 10%, or 15% F fermented Mao-tai lees, respectively

**Fig. 3:**
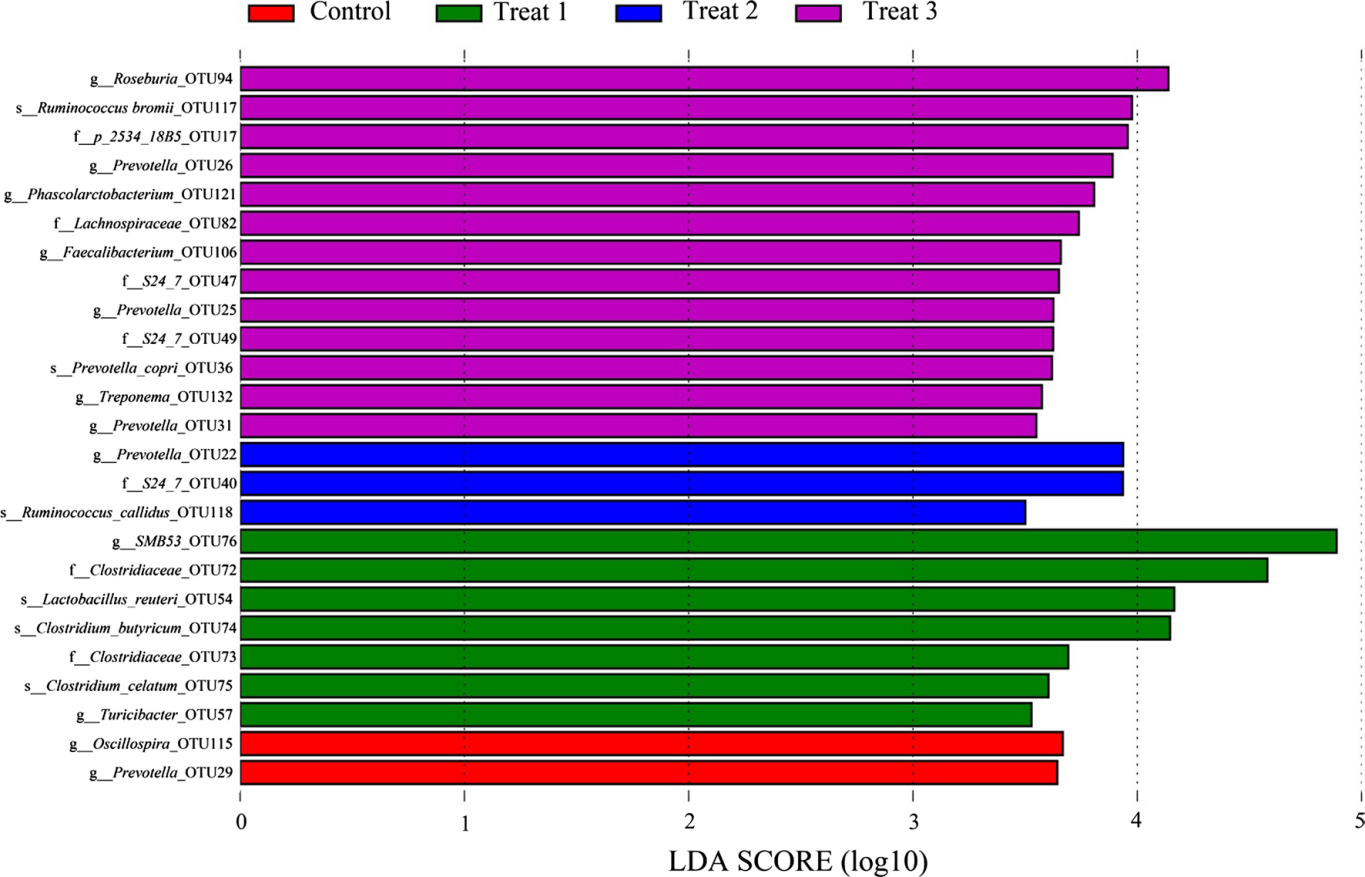
Differences in the pig gut microbiota among the different fermented Mao-tai lees (FML) treatments. Linear discriminant analysis (LDA) effect size (LEfSe) results show that bacterial OTUs/markers were significantly different in abundance between control and FML-treated groups

To further compare the taxonomic difference between the control and treat groups, we used LEfSe analysis to differential abundance of bacterial taxa at OTU level among different treatments (Fig. 3). The results showed that a total of 25 bacterial biomarkers/OTUs were differentially abundant among the four groups. OTU54 (belonging to *Lactobacillus reuteri*), OTU57 (*Turicibacter*), OTU73 (Clostridiaceae), OTU72 (Clostridiaceae), OTU74 (*Clostridium butyricum*), OTU75 (*Clostridium celatum*), and OTU76 (*SMB53*) were the dominant microbes in the 5% FML group, while OTU 29 (*Prevotella*) and OTU115 (*Oscillospira*) were mostly in the control group. A total of 13 OTUs, including OTU117 (*Ruminococcus bromii*), OTU121 (*Phascolarctobacterium*), OTU132 (*Treponema*), OTU 25, 26, and 31 (*Prevotella*), OTU106 (*Faecalibacterium*), OTU94 (*Roseburia*), and OTU36 (*Prevotella copri*), were significantly enriched in the 15% FML group.

### Comparison of the potential beneficial bacteria and pathogens abundance

**Fig. 4:**
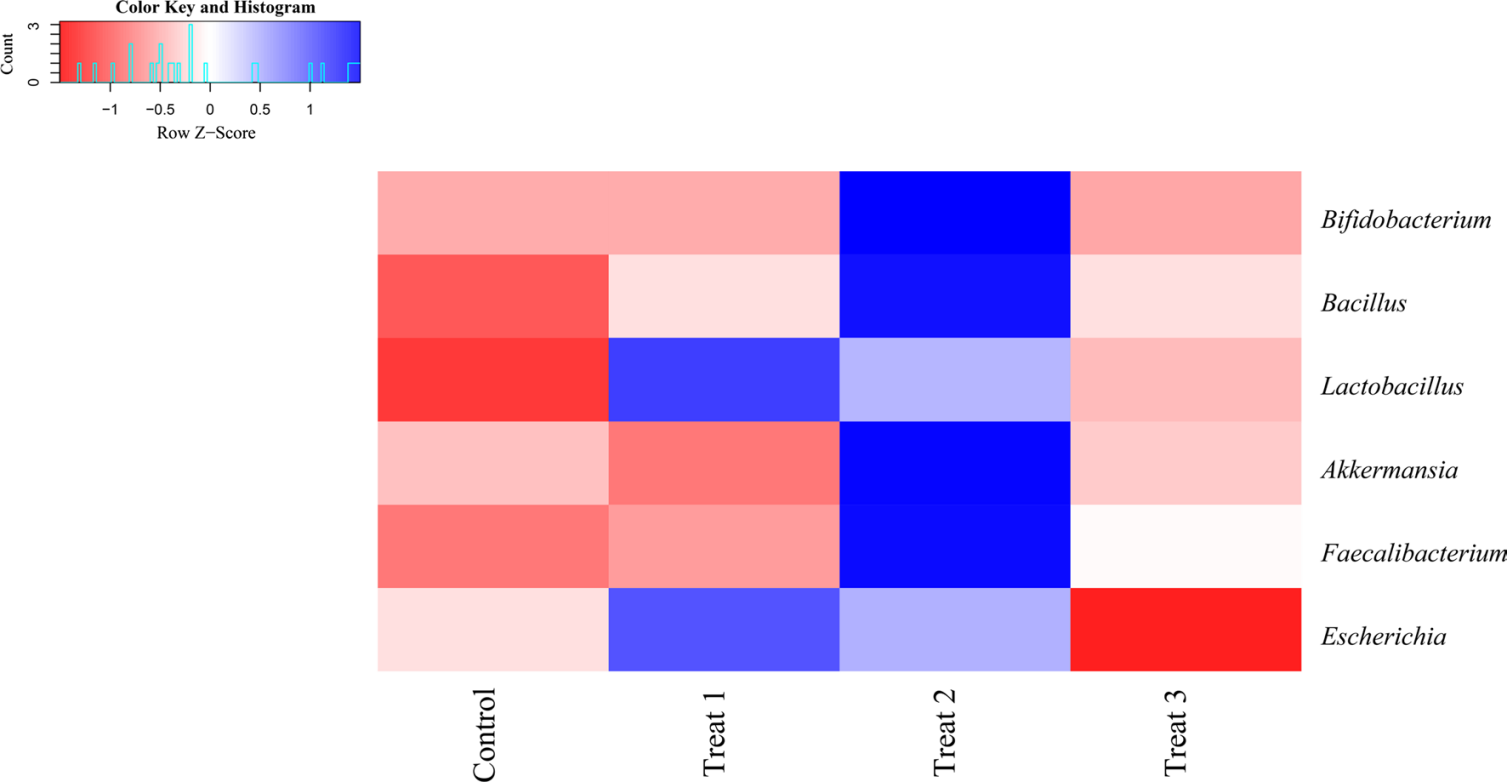
The distribution of potential probiotics and pathogens of pigs among treatments. The mean relative abundance of these genera was normalized using Z-score transformation

In order to detect the effects of FML on the potential probiotics (*Bifidobacterium*, *Bacillus*, *Lactobacillus*, *Akkermansia*, and *Faecalibacterium*) and pathogens (*Escherichia*) in pigs (Barba-Vidal et al. 2018; Rastall and Gibson 2015), we compared the abundance of these genera among treatments (Fig. 4). We found that the relative abundance of *Bacillus*, *Lactobacillus*, *Akkermansia*, and *Faecalibacterium* in 15% FML group is higher than that of the control group. However, the 15% FML showed a lower abundance of *Escherichia* than that of the control group.

### The differences of predicted gene functions across groups

Based on the functional prediction of 16S rRNA gene sequences, the difference of gene functions among groups were visualized based on NMDS plot of Bray–Curtis dissimilarity at level 3. Overall functional profiles showed no significant differences among groups (PERMANOVA, *R*^2^ = 0.197, *P* > 0.05, Additional file 1: Fig. S2). When we compared the differences between the 15% FML and control groups, a total of 29 gene functions at level 3 showed significant differences between the two groups. Compared with the control group, the 15% FML was more abundant in 15 gene functions associated with metabolism, such as flavone and flavonol biosynthesis, phenylpropanoid biosynthesis, starch and sucrose metabolism, carbon fixation in photosynthetic organisms, sulfur metabolism, nicotinate and nicotinamide metabolism, retinol metabolism, and cyanoamino acid metabolism (Additional file 1: Table S3).

### Correlations between bacterial biomarkers and gut metabolites

**Fig. 5:**
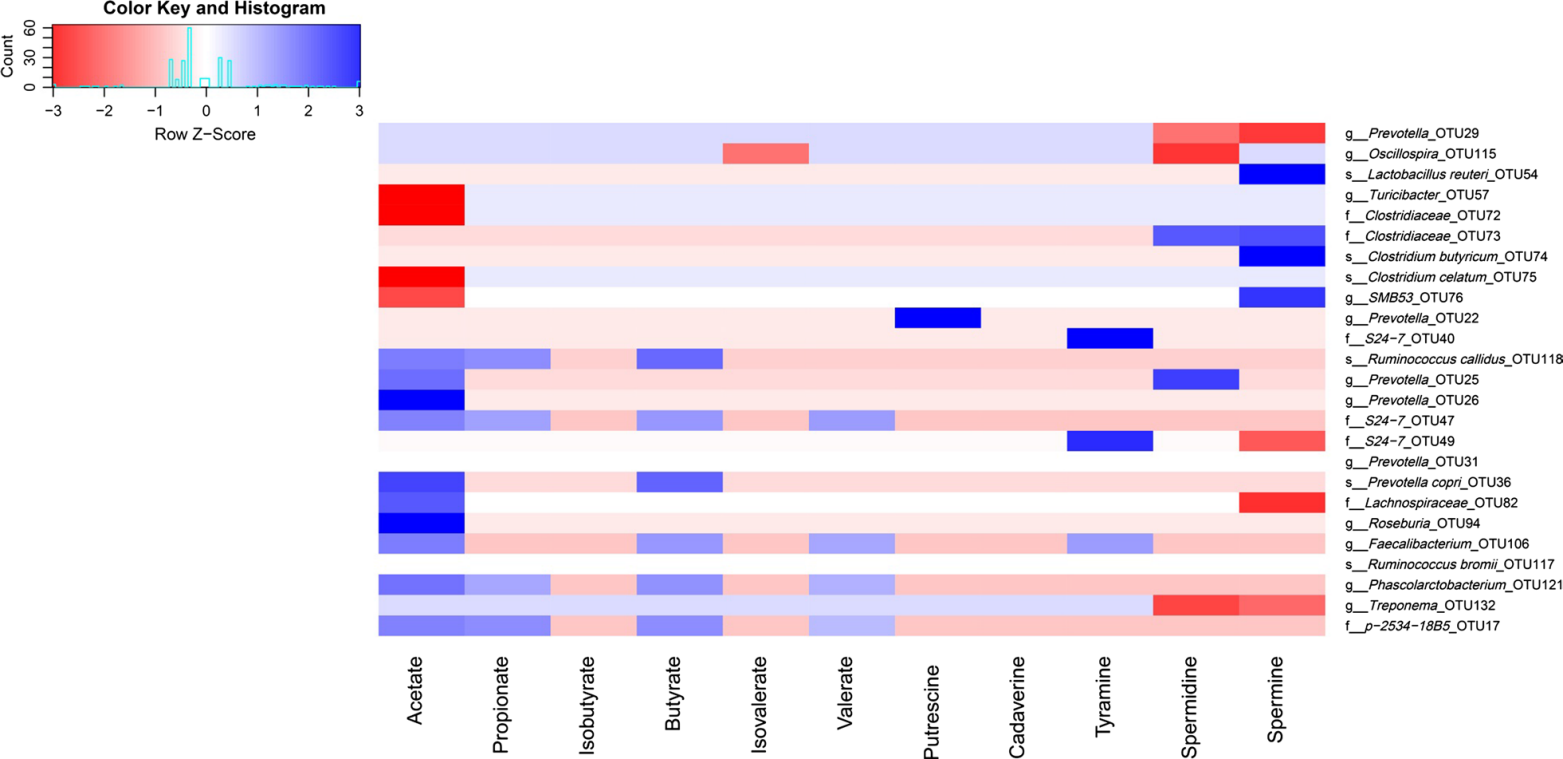
The heatmap plot of spearman correlations between bacterial markers and SCFAs or bioamines. Only those correlations with *P* < 0.05 are shown

To understand the relationship between bacterial biomarkers and gut metabolites, we calculated the spearman correlations between those bacterial OTUs (based on LefSe analysis) and SCFAs or bioamines (Fig. 5). Our results showed that acetate was negatively correlated with OTU57, OTU72, OTU75, or OTU76, whereas was positively associated with OTU17, OTU25, OTU26, OTU36, OTU47, OTU82, OTU94, OTU106, OTU118, and OTU121. Propionate only showed positive correlations with OTU17, OTU47, OTU118, and OTU121. Butyrate was related to OTU17, OTU36, OTU47, OTU106, OTU118, and OTU121. These results indicated that most OTUs enriched in the 15% FML group showed correlations with SCFAs. In addition, OTU22 was positively correlated with Putrescine. Tyramine showed positive associations with OTU40, OTU49, and OTU106.

## Discussion

In the field of animal nutrition, the development of new potential prebiotics in animal husbandry industries is of current interest. Recently, high-throughput sequencing of 16S rRNA gene amplicons has been used to investigate potential beneficial effects of various feed additives (e.g., xylo-oligosaccharides and yeast cultures) on the gut microbial communities of farmed animals (Liu et al. 2018; Pourabedin et al. 2015). However, relatively few studies focused on the effects of liquor lees on the composition and function of livestock gut microbiota. This study is the first to report the influence of FML on pig gut microbiota and metabolic profiles. Our results indicated that dietary supplementation with 15% FML significantly influenced gut microbial community structure and bacterial metabolite concentrations in pigs, and those abundant OTUs in the 15% FML group were positively associated with the fermentation of dietary fiber. Moreover, 15% FML improved the relative abundance of potential beneficial bacteria and decreased the abundance of pathogens. These results greatly enhanced our understanding of the potential beneficial effects of FML on pigs.

### FML improve the fermentation ability of gut microbiota for dietary fiber in pigs

Animals acquire nutrients and energy via bacterial fermentation of ingested feed. SCFAs that are major end metabolic products result from the bacterial fermentation of dietary fiber (including cellulose, lignin, and other polysaccharides) in mammalian guts (den Besten et al. 2013). Our results showed that higher FML content in feed were associated with increased concentrations of straight-chain fatty acids, including acetate, propionate, butyrate, and valerate, indicating that FML improve the amount of these SCFAs in the colonic contents. These SCFAs play pivotal roles in several host physiological functions, including nutrient utilization, energy expenditure, gut immunity, and macromolecular synthesis (Koh et al. 2016). For example, butyrate provides 60–70% energy source for colonic epithelial cells and also protects against colorectal cancer and inflammation (Flint et al. 2012; Scheppach 1994). Butyrate-enriched high-fat diet in mice increased thermogenesis and energy expenditure, and the hosts are resistant to obesity (Gao et al. 2009). Acetate entering peripheral circulation can be metabolized by peripheral tissues and then transported to the liver for cholesterol synthesis (Wolever et al. 1989). Acetate dietary supplements in obese and diabetic rats improve glucose tolerance and reduce weight gain of animals (Yamashita et al. 2014). Most propionate is absorbed by the liver and is a good precursor for gluconeogenesis, liponeogenesis, and protein synthesis (Wolever et al. 1991). Food containing propionate in healthy women increased insulin release and reduced the fasting glucose level (Venter et al. 1990). These straight-chain fatty acids are indicators of the fermentation of dietary fiber, thus FML possibly improve the fermentation ability of gut microbiota for dietary fiber.

BCFAs (e.g., isobutyrate and isovalerate) are metabolites that result from protein fermentation via gut bacteria (Birkett et al. 1996), and isobutyrate and isovalerate originate from l-leucine and l-valine, respectively (Le et al. 2005). BCFA amounts in the gut contents could be regarded as indicators of protein catabolism in the colon (Blachier et al. 2007). Our results showed that FML additive amounts were not associated with BCFAs, indicating that FML supplements likely failed to improve protein catabolism. Thus, our results indicate that the increased SCFA net concentration in the colon is likely the result of increased fermentation of specific dietary fibers by intestinal microbiota.

Although FML improve the concentrations of straight-chain fatty acids, higher gut SCFA concentrations in 15% FML could result from increased SCFA production or decreased SCFA absorption in the colon, or the fewer bacterial species in the gut utilize the SCFAs as an energy source. SCFA production and profiles in humans or animals were regulated by a lot of different host traits, environmental, dietary or microbial factors (Li et al. 2018; Macfarlane and Macfarlane 2003). The FML groups had higher contents of crude fiber and protein than the control group (Additional file 1: Table S1), thus one possible factor that controls specific SCFA production in our study is possibly nutrient components of available feed. We found that there are several OTUs that were significant different between the control and 15% FML groups and most of these OTUs were positively with the straight-chain fatty acids, thus gut microbiota composition may be another important factor that influences specific gut SCFA. Overall SCFA profile structure was significant influenced by body weight rather than by FML (Fig. 1), indicating that body weight may regulate the relative percentage of these SCFAs. In addition, those unmeasured factors, such as gut transit time, can also cause the difference of SCFA concentration (Cummings 1978) among different groups. Therefore, this may result in a limited understanding for the relationship between food fermentation time and SCFAs.

Bioamines are microbial fermentation products and are derived from amino acids (AA) metabolism. Bioamines can be produced based on AA decarboxylation pathways by specific bacteria (Tuberoso et al. 2015). Bioamines play important roles in various bacterial functions, such as maintaining growth and reproduction of normal cells and reducing bacterial susceptibility to host-derived antimicrobials (Shah and Swiatlo 2008). In addition, bioamines are correlated with numerous host diseases (e.g., psychiatric and neurologic disorders) (Stahl 1977), thus may be an indicator of host immunity and health. However, our results that most of bioamines were not correlated with FML content in feed, indicating that FML may have no impacts on the bioamines. Notably, tyramine was positively correlated with FML content. The possible reason is that FML improve the relative abundance of some tyramine-producing bacteria, such as *Lactobacillus* (De Las et al. 2006) which enriched in those FML groups.

### FML influence the beta diversity and metabolism-associated gene functions of pig gut microbiota

Our results showed that FML had no significant impacts in the alpha diversity, indicating that microbial species diversity is relatively stable in response to FML. However, FML influenced the beta diversity (Jaccard and Bray–Curtis dissimilarity) of pig gut microbiota compared with the control group, indicating that FML may influence species replacement (changes in species taxa) and species sorting (changes in abundance). Notably, only 15% FML was able to shape the community structure compared with the control group. These results suggested that the effect of FML on gut microbiota structure depends on additive amount. One possible reason is that a high-content dietary fiber in the 15% FML modified the gut microbiota structure, as demonstrated by previous study (Tap et al. 2015). However, our data showed that overall gene function profiles showed no difference among groups, indicating that overall functions of gut microbiota were more stable possibly for maintaining the gut performance. Compared with the control group, the 15% FML was enriched in some gene functions associated with metabolism (e.g., starch and sucrose metabolism and sulfur metabolism), thus 15% FML not only influence the gut microbiota structure but also function. In addition to dietary fiber, other nutrients, such as protein, may also impact the structure and function of gut microbiota (Scott et al. 2013).

### FML increase the abundance of several potential beneficial bacteria but decrease the abundance of specific pathogens

Our results showed that the phyla Firmicutes and Bacteroidetes accounted for 89.1% of total sequences, similar to previous findings in the microbiota of large intestines and feces in pigs (Bian et al. 2016; Kelly et al. 2017; Mach et al. 2015). At genus level, we found that *Prevotella* was the most dominant bacterial genera in the pig gut. *Prevotella* is known to produce various enzymes, such as glycoside hydrolases and polysaccharide lyase enzymes (Kaoutari et al. 2013), and the abundance changes of this genus is associated with dietary fiber (Liu et al. 2012). Thus, a higher abundance of *Prevotella* in the 15% FML may result from higher fiber content for 15% FML, so that the fiber ingredients improve the enrichment of this genus. *Prevotella* was also abundant in the gut microbiota from Burkina Faso children, whose diet ingredients contain a large amount of plant fiber (De Filippo et al. 2010). In fact, it has been demonstrated that *Prevotella* was capable of degrading plant cell wall dietary fiber, and thus may produce a great deal of SCFAs that can be utilized by hosts and microbes (De Filippo et al. 2010). In addition to *Prevotella*, *Roseburia* was also enriched in the 15% FML group. *Roseburia* is butyrate-producing bacteria (Machiels et al. 2014), thus a higher abundance of this genus may lead to higher concentration of butyrate in the 15% FML group.

LEfSe analysis offered high-resolution discrimination at OTU level, and the results showed that those OTUs belonging to *Prevotella* (OTU25, OTU26, OTU31, and OTU36) and *Roseburia* (OTU94), *Ruminococcus bromii* (OTU117), *Treponema* (OTU132), *Phascolarctobacterium* (OTU121), and *Faecalibacterium* (OTU106), were significantly enriched in the 15% FML group. Some members of *Ruminococcus* and *Treponema* are involved in cellulose, lignin, and resistant starch degradation (Niu et al. 2015; Ze et al. 2012). *Faecalibacterium* is one of the most important commensal bacteria in the human gut microbiora. Several members of *Faecalibacterium*, as well as *Phascolarctobacterium* may produce various SCFAs (including acetate and butyrate) through the fermentation of dietary fiber (Lukovac et al. 2014), as demonstrated by our data, which found that most of these abundant OTUs in the 15% FML group were positively correlated with acetate and butyrate. However, it is still difficult to determine which bacteria taxa were responsible for the specific SCFA due to the complex interactions among bacteria, such as cross-feeding (Rey et al. 2010) and resource competition (Mahowald et al. 2009).

Accumulative evidence has demonstrated that some potential prebiotics may stimulate the growth of beneficial bacteria and be resistant against pathogenic bacteria. In this study, we compared the relative abundance of five potential probiotis and one pathogen between the control and treat groups. Our results showed that the potentially beneficial bacterial genera *Bacillus*, *Lactobacillus*, *Akkermansia,* and *Faecalibacterium* were more abundant in the 15% FML, while the potentially pathogenic genera *Escherichia* had a lower abundance. Some members of *Bacillus,* such as *Bacillus subtilis,* may reduce diarrhea scores and modulate microbial diversity in pigs (Bhandari et al. 2008). *Lactobacillus* provided many beneficial effects for pigs, such as improving ileal histomorphology, reducing systemic inflammatory cytokines, and increasing fermentation ability (Guerra-Ordaz et al. 2014). *Akkermansia*, especially *Akkermansia muciniphila*, is a mucin-degrading bacterium derived from mucus layer and shows a positive correlation with metabolic ability and health in mice (Everard et al. 2013). Some reports found that an increase in abundance on *Faecalibacterium* and *Akkermansia* was associated with a healthy gut status. In contrast, *Escherichia* is one known gut pathogens, and can lead to various diseases, such as diarrhea (Sack 1975). These results demonstrated that 15% FML had beneficial effects on the balance of pig gut microbiota.

In conclusion, this study is the first time to investigate the beneficial effects of FML on gut microbiota and metabolic profiles in pigs. Our results clearly demonstrated that 15% FML modulated the gut microbiota structure and function. Importantly, 15% FML increased the abundance of putative beneficial bacteria and decreased the abundance of potential pathogen *Escherichia* spp. in the pig gut, and also contributed to improve the fermentation of dietary fiber or indigestible polysaccharides in feed. Consequently, our results indicated that FML may be developed as a new feed additive for healthy and ecological livestock farming.

## Additional file

**Additional file 1.** Additional tables and figures.
