# Supplementary material for: Dietary supplementation with fermented Mao-tai lees beneficially affects gut microbiota structure and function in pigs
Source: AMB Express. 2019 Feb 18;9:26. doi: 10.1186/s13568-019-0747-z (PMC6379501; doi:10.1186/s13568-019-0747-z)
Supplement: Supplementary file 1 — Additional file 1. Additional tables and figures. [file 13568_2019_747_MOESM1_ESM.pdf]

Submitted to *AMB express*

Dietary supplementation with fermented Mao-tai lees beneficially affects gut microbiota structure and function in pigs

Huan Li[1, 2$], Huawei Li[1$], Peifeng Xie[1], Zhihua Li[1], Yulong Yin[1], Francois Blachier[3], Xiangfeng Kong[1*]

1 Hunan Provincial Key Laboratory of Animal Nutritional Physiology and Metabolic Process, Key Laboratory of Agro-ecological Processes in Subtropical Region, National Engineering Laboratory for Pollution Control and Waste Utilization in Livestock and Poultry Production, Institute of Subtropical Agriculture, Chinese Academy of Sciences, Changsha, Hunan 410125, China

2 Institute of Occupational Health and Environmental Health, School of Public Health, Lanzhou University, Lanzhou, Gansu 730000, China

3 UMR PNCA, AgroParisTech, INRA, Université Paris-Saclay, Paris, France

[$] These authors contributed equally to this work

Author E-mail information:

Huan Li: lihuanzky@163.com; Huawei Li: 463586800@qq.com; Peifeng Xie: 1411575007@qq.com; Zhihua Li: 864506798@qq.com; Yulong Yin: yinyulong@isa.ac.cn; Francois Blachier: Francois.Blachier@agroparistech.fr; Xiangfeng Kong: nnkxf@isa.ac.cn

* Corresponding author, Xiangfeng Kong, E-mail: nnkxf@isa.ac.cn

Running title: Fermented Mao-tai lees and gut microbiota

Table S1. Composition and nutrient levels of the basal diets (air-dry basis).

Ingredient, %	Growing phase (45 to 75 kg)	Finishing phase (75 to 110 kg)
Corn	59.49	59.66
Barley	8.00	8.00
Soybean oil	1.50	1.00
Soybean meal	25.00	25.50
$CaHPO_4$	0.10	—
Calcium carbonate	1.08	1.08
Salt	0.43	0.43
Lys	0.18	0.13
Met	0.03	—
Thr	0.07	0.08
Antioxidants	0.02	0.02
Antimildew agent	0.10	0.10
Premix[1]	4.00	4.00
Total	100.00	100.00
Nutrient levels[2]		
Digestible energy (DE, MJ/kg)	13.78	13.65
Crude fiber (CF)	2.86	2.89
Crude protein (CP)	16.40	16.50
Ether Extract (EE)	4.30	3.80
Lys	1.08	1.05
Met	0.30	0.28
Thr	0.71	0.73
Ca	0.74	0.66
Total P	0.52	0.45

1 Premix provided for 1 kg of complete diet: Cu as copper sulfate,10 mg; Fe as iron sulfate, 100 mg; Se as sodium selenite, 0.30 mg; Zn as zinc oxide, 100 mg; Mn as manganese oxide, 10 mg; vitamin D_3, 386 IU; vitamin A as retinyl acetate, 3 086 IU; vitamin E as D-atocopherol, 15.4 IU; vitamin K as menadione sodium bisulfate, 2.3 mg; vitamin B_2, 3.9 mg; calcium pantothenate, 15.4 mg; niacin, 23 mg; and vitamin B_{12}, 15.4 mg.

2 Nutrient contents were calculated according to their contents in the basal diet group.

Table S2. PERMANOVA showing different short chain fatty acid (SCFA) and bioamine profiles among groups based on Bray-Curtis dissimilarity.

Items	SCFA		Bioamine	
	R^2	*P*	R^2	*P*
All	0.151	0.338	0.232	0.106
Control vs Treat 1	0.086	0.433	0.174	0.157
Control vs Treat 2	0.06	0.522	0.163	0.187
Control vs Treat 3	0.157	0.139	0.268	0.073
Treat 1 vs Treat 2	0.109	0.328	0.00039	0.982
Treat 1 vs Treat 3	0.051	0.59	0.078	0.478
Treat 2 vs Treat 3	0.245	0.051	0.113	0.307

No significant differences ($P < 0.05$) are observed. The group control, treat 1, treat 2, and treat 3 signify that feed is supplemented with 0, 5%, 10%, or 15% FML, respectively.

Table S3. The difference of predicted gene functions at level 3 between the treat 3 and control group.

Gene functions (mean abundance)	Control	Treat 3	*P* value
Environmental Information Processing; Signaling Molecules and Interaction; G protein-coupled receptors	0.000002	0.000000	0.033
Genetic Information Processing; Replication and Repair; Chromosome	0.0164	0.0170	0.041
Human Diseases; Immune System Diseases; Primary immunodeficiency	0.0004	0.0005	0.021
Human Diseases; Immune System Diseases; Systemic lupus erythematosus	0.0000	0.0000	0.049
Human Diseases; Neurodegenerative Diseases; Huntington's disease	0.0003	0.0002	0.008
Metabolism; Amino Acid Metabolism; Tryptophan metabolism	0.0015	0.0013	0.012
Metabolism; Biosynthesis of Other Secondary Metabolites; Flavone and flavonol biosynthesis	0.0000	0.0001	0.012
Metabolism; Biosynthesis of Other Secondary Metabolites; Phenylpropanoid biosynthesis	0.0014	0.0017	0.021
Metabolism; Carbohydrate Metabolism; Butanoate metabolism	0.0067	0.0061	0.038
Metabolism; Carbohydrate Metabolism; Propanoate metabolism	0.0052	0.0047	0.036
Metabolism; Carbohydrate Metabolism; Starch and sucrose metabolism	0.0095	0.0104	0.012
Metabolism; Energy Metabolism; Carbon fixation in photosynthetic organisms	0.0064	0.0068	0.020
Metabolism; Energy Metabolism; Sulfur metabolism	0.0024	0.0026	0.033
Metabolism; Metabolism of Cofactors and Vitamins; Nicotinate and nicotinamide metabolism	0.0043	0.0047	0.047
Metabolism; Metabolism of Cofactors and Vitamins; Retinol metabolism	0.0001	0.0002	0.011
Metabolism; Metabolism of Other Amino Acids; Cyanoamino acid metabolism	0.0027	0.0030	0.046
Metabolism; Metabolism of Other Amino Acids; Phosphonate and phosphinate metabolism	0.0006	0.0005	0.046
Metabolism; Metabolism of Terpenoids and Polyketides; Biosynthesis of siderophore group nonribosomal peptides	0.0001	0.0002	0.038
Metabolism; Metabolism of Terpenoids and Polyketides; Limonene and pinene degradation	0.0009	0.0008	0.028
Metabolism; Xenobiotics Biodegradation and Metabolism; Aminobenzoate degradation	0.0011	0.0010	0.042
Metabolism; Xenobiotics Biodegradation and Metabolism; Atrazine degradation	0.0002	0.0001	0.036
Metabolism; Xenobiotics Biodegradation and Metabolism; Ethylbenzene degradation	0.0005	0.0004	0.030
Organismal Systems; Digestive System; Carbohydrate digestion and absorption	0.0001	0.0002	0.023

Organismal Systems; Endocrine System; Insulin signaling pathway	0.0007	0.0009	0.028
Organismal Systems; Excretory System; Proximal tubule bicarbonate reclamation	0.0002	0.0002	0.019
Organismal Systems; Nervous System; Glutamatergic synapse	0.0010	0.0010	0.002
Unclassified; Metabolism; Amino acid metabolism	0.0021	0.0017	0.007
Unclassified; Metabolism; Carbohydrate metabolism	0.0013	0.0015	0.005
Unclassified; Metabolism; Lipid metabolism	0.0014	0.0015	0.019

The group control and treat 3 signify that feed is supplemented with 0 or 15% FML, respectively.

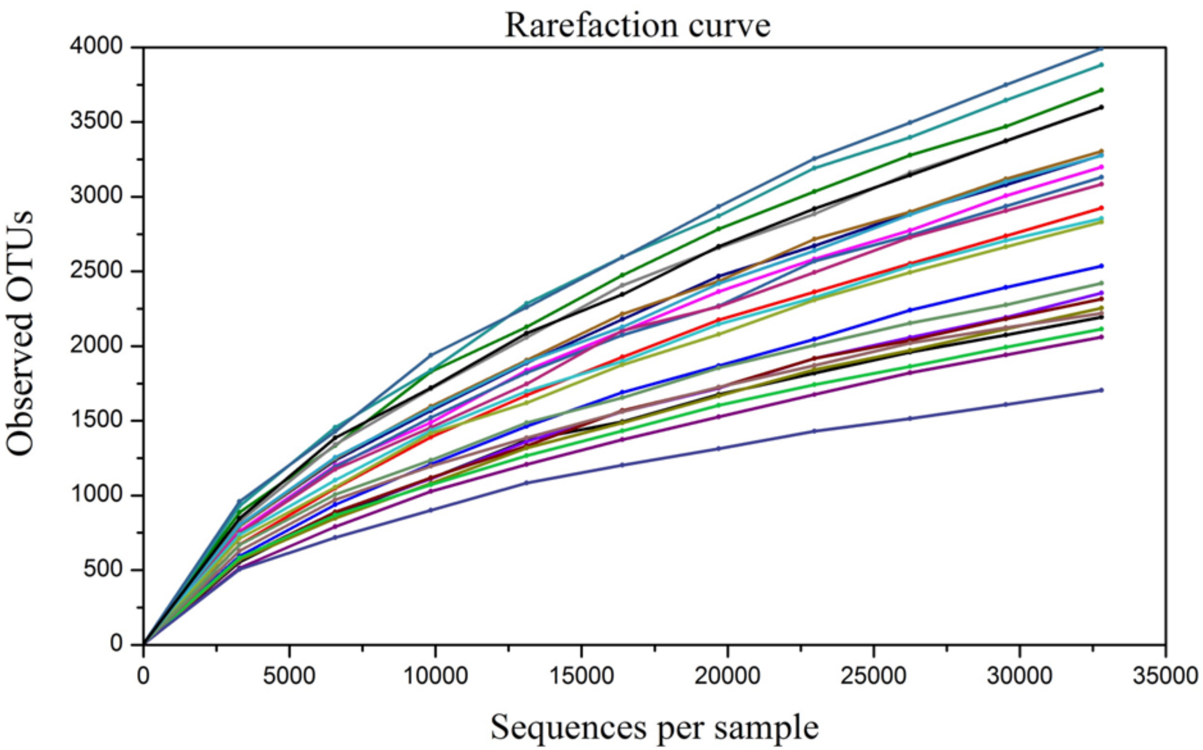

Fig. S1. The rarefaction curve of observed OTUs across all samples

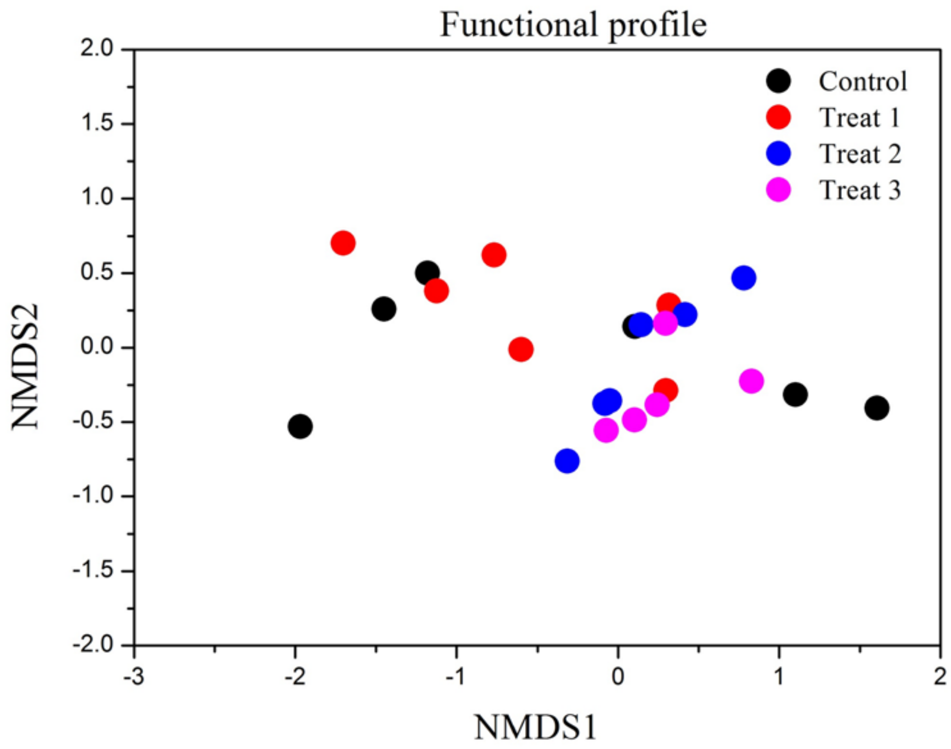

Figure S2. The NMDS plot showing the difference of predicted gene functional profile at level 3 based on the Bray-Curtis dissimilarity among groups.
